# Chemotherapy in conjoint aging-tumor systems: some simple models for addressing coupled aging-cancer dynamics

**DOI:** 10.1186/1742-4682-7-21

**Published:** 2010-06-15

**Authors:** Mitra S Feizabadi, Tarynn M Witten

**Affiliations:** 1 Physics Department, Seton Hall University, South Orange, NJ07079, USA; 2 Center for the Study of Biological Complexity, Virginia Commonwealth University, Richmond, VA 23284-2030, USA

## Abstract

**Background:**

In this paper we consider two approaches to examining the complex dynamics of conjoint aging-cancer cellular systems undergoing chemotherapeutic intervention. In particular, we focus on the effect of cells growing conjointly in a culture plate as a precursor to considering the larger multi-dimensional models of such systems. Tumor cell growth is considered from both the logistic and the Gompertzian case, while normal cell growth of fibroblasts (WI-38 human diploid fibroblasts) is considered as logistic only.

**Results:**

We demonstrate, in a simple approach, how the interdependency of different cell types in a tumor, together with specifications of for treatment, can lead to different evolutionary patterns for normal and tumor cells during a course of therapy.

**Conclusions:**

These results have significance for understanding appropriate pharmacotherapy for elderly patients who are also undergoing chemotherapy.

## Prologia

In 1976 I (TMW) attended a small meeting at the W. Alton Jones Cell Science Center, a research center in upstate New York. I was a young graduate student and one of the presenters was a then very young James Smith. He presented a talk on WI-38 human diploid fibroblast doubling and aging [1]. The results of his work lead to clonal fibroblast data distributions that looked surprisingly similar to my Master's degree modeling work on recombination of tandem gene repeats and their possible relationship to aging and cancer [2,3]. I was immediately addicted to trying to model the processes of aging in normal cells. Not that long afterwards, I attended a cancer conference and two presenters, Leonard Weiss and Robert Kerbel, grabbed my attention talking about cancer metastasis. For me, now intrigued by biomedical aging processes, the obvious question was "how does aging change metastasic processes?" Despite what I thought were some rather elegantly designed experiments put forth in grant proposals designed to study this question in mice, the American Cancer Society felt that the topic was not relevant and that I - a mathematical physicist - was far from qualified to perform said proposed experiments. They were quite correct on the latter and far from correct on the former.

Despite my initial failures with the ACS grants, I felt quite committed to trying to develop a mathematical model of normally aging fibroblast cells. Models of cancer cells and cancer cell population behavior abounded, but nowhere could I find a model that described cellular aging [4,5]. Thus began a decade of research papers [6-9] culminating in a series of cellular aging modeling developments [10,11] that were eventually laid to rest due to lack of ability to obtain the experimental data needed to expand and validate the models. In parallel, I also developed a series of models attempting to describe the interplay of aging normal fibroblasts and tumor cells [6,12-14].

Not long after the retirement of this research effort, I was asked to contribute to a special issue of the Journal of Gerontology on the subject of aging and cancer. That paper, Witten (1986) [13] presented the first simple ordinary differential equation model of conjoint tumor-normal cell growth, demonstrating that it was - in fact - possible to obtain different joint cellular stability configurations for the two cell populations, depending upon how the cells talked with each other through the set of rules defining inter-cellular communication. We begin by asking the following question: Why study the aging-cancer question?

## The Aging-Cancer Question

### Demographics of Aging

In the United States, more than 13 percent of the total population is over the age of 65, representing one in every eight Americans [15]. The majority of these older people are women, representing almost 60 percent of the elderly population [15]. More than half of this population falls in Hooyman & Kiyak's classification of young old; 53 percent are between 65 and 74 years of age. While the oldest old (85 years old and over) represent only 12% of this group, this is the fastest-growing demographic group in the United States [16]. People of ethnic minority status represented only 16 percent of the elderly population in 1998, yet this is rapidly changing. By the year 2050, more than 30 percent of the older Americans will be those who are not primarily of European ancestry, including 16 percent Hispanics, 10 percent African Americans, 7 percent Asian and Pacific Islanders, and 1 percent Native Americans, according to current estimates [15].

Poverty is a major concern for all older Americans, particularly in the light of recent increases in the cost of health care, including medications. Lack of comprehensive health care contributes to increased levels of poverty among the old. More than half of elderly persons report living with at least one disability. The poverty rate is doubled among those whose disability affects their mobility or their ability to take care of themselves [16]. The implication here is that many of these individuals cannot afford their own medications much less treatment for cancer.

Based on the federal poverty guidelines, 11 percent of the old live in poverty, with another 6 percent living near poverty levels, with incomes just 25 percent higher than the poverty line [16]. Twenty-six percent of African American and 21 percent of Hispanic elderly persons live in poverty [15]. These figures may not offer a complete picture of the socioeconomic state for most of the old in the United States. AARP states that 40 percent of all older people in the United States live on incomes less than 200 percent of the poverty level [15].

Nearly twice as many older women than older men live in poverty: 13 percent versus 7 percent. Older members of minority groups and those who live alone also experience a higher risk of poverty [15,16]. Twenty percent of older persons who live alone are poor. Almost half of old women (42%) live alone, as opposed to old men (20%), resulting in higher poverty rates among women. This discrepancy is more pronounced among members of many ethnic minorities, because the life expectancy of men is proportionately lower [16]. Thirteen percent of white (European American) women who live alone live in poverty. Almost half (49%) of African American women who live alone are living below the poverty level [15]. It is estimated that without Social Security, the elderly poverty rate would soar to 54 percent [15,16].

The preceding portion of this discussion has focused on Western nations, while including some salient facts about global aging [17-29]. In 2000, there were 600 million people aged 60 and over in the world [30]. The World Health Organization estimates that there will be 1.2 billion people aged 60 and over by 2025 and 2 billion by 2050. Today, about 66% of all older people are living in the developing world; by 2025 it will be 75%. As of 1 July 2004, there were 36.3 million people in the US, over the age of 65, 4.8 million people over the age of 85, and 64,658 people estimated to be 100 years old or over on 1 August 2004. It is projected that there will be 86.7 million people in the US, over the age of 65 in the year 2050, comprising 21% of the total US population at that time. This will represent a 147% increase in the 65 years old and over population in the United States between 2004 and 2050.

In terms of percent of population aged 65 and over, the US is young in comparison to the rest of the developed world. With the exception of Japan, the world's 25 oldest countries (as of 2001) are all in Europe (see Figure 2, 3 of [22]. Projections of the monthly gain of individuals age 65 and over, to the year 2010, are as large as 847,000 people per month worldwide. In 2000, 615,000 of the world's net gain of elderly individuals per month occurred in developing countries [22]. Projections for Europe indicate that by 2015, the percentage of over 65-year old individuals will be the greatest and by 2030, nearly 12% of all Europeans are projected to be over the age of 74 and 7% are projected to be over the age of 79. Levels in Asia, Latin America/Caribbean are expected to more than double by 2030, while aggregate proportions of elderly in the Sub-Saharan Africa are projected to grow modestly as a result of continued high fertility in many nations [22]. However, in the developed world, the very old (ages 80 and older) is the fastest growing population sub-component [29]. Given these trends, late life and end of life care will become increasingly important in the decades ahead [31]. As part of this lifecare, cancer therapy will become a more and more important component as the global population continues to age.

### Demographics of Aging and Cancer

In 1974 Burnet [32] published data which illustrated an age-specific exponential increase in certain human cancers; stomach cancer in males, breast cancer in females. Pitot [33] also addressed aging and carcinogenesis. His Table [1] provides an excellent comparison between neoplasia and aging factors; reinforcing the variety of similarities between the two processes. In 1981, Cohen *et al. *[34] show much the same results for the incidence of hematologic tumors in humans. A 1982 Oncology Overview [35] cites 192 abstracts of papers discussing the age-related factors which may predispose to carcinogenesis. In that same year Weindruch & Walford [36] pointed out that lifelong dietary restriction, beginning at 3-6 weeks of age in rodents is known to decelerate the rate of aging, increase mean and maximum lifespans and to inhibit the occurrence of many spontaneous tumors. DeVita [37] contains some 33 papers discussing issues that impinge on the age-related incidence of various types of cancer. Ebbesen [38] discusses the probable mechanisms of cancer development and "those aspects of 'normal' aging that he believes to be most relevant to the etiologic and pathogenetic bonds between the two biological processes." These mechanisms are explored in Macieria-Coelho & Azzarone [39]. Mathe & Reizenstein [40] further discuss the aging-cancer relationship in humans. They point out that incidence of many tumors (most of the carcinomas and leukemias) increases with age; for a combination of reasons. Among these reasons are environmental factors, decreased DNA repair function, decreased immunological and biological surveillance for tumors, and a lack of hormonal regulation. The incidence rates are seen to rise sharply, once one is past the age of thirty, with a dramatic increase once one is past the age of fifty.

**Figure 1:**
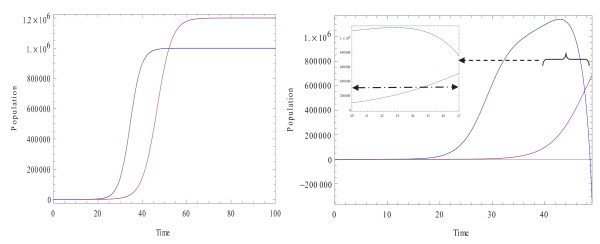
**Blue curve: Evolution of normal cells**. **Purple curve: **Evolution of tumor cells. Common parameters: *r*_*N *_= 0.4, *r*_*T *_= 0.3, *K*_*T *_= 1.2.10^6^, *K*_*N *_= 10^6^. **Left: **There is no interaction between normal cells and tumor cells (both populations undergo logistic growth), *k *= 0, *β *= 0. **Right: **Normal and tumor cells are allowed to interact with each other, *k *= 1, *β *= 2, *ρ*_0 _= 1, *ρ*_1 _= 1000, T* = 3.10^5^, *N*_0 _= 1, *T*_0 _= 1. The mini-window magnifies the behavior of normal and tumor cells close to the critical size of the tumor. As the size of the tumor cells *T *exceed the critical size, T* (dashed line), the size of normal cells *N *starts decreasing.

More recently, DePinho [41] points out that "a striking link exists between advanced age and increased incidence of cancer" and that "aging is the most potent of all carcinogens." He points out that the incidence of invasive cancer, when plotted against age, reveals exponential increases from ages 40-80 years old [41] (Figure [1]. More recently, Yanic & Ries [42] point out that cancer in older persons is an international issue that needs to be addressed. How might the processes of aging and cancer be interrelated?

## Cellular Aging and Cancer

### Age-related Cancer Treatment

As we age, our bodies change in numerous ways. Biomedical dynamics is altered, metabolism slows, organ function can diminish in conjunction with an increase in the number of prescription drugs taken. Liver and kidney function can change making clearance rates for drugs change and potentially increasing the chances of multi-drug interactions that could be harmful or even fatal. The body's ability to withstand toxins [43] often decreases making it potentially more difficult to treat various forms of cancer with cytotoxic agents [44-46]. Pharmacological considerations must also be taken into account, not only from the perspective of which is the optimal chemotherapeutic agent and at what toxicity level, but also one must consider what other drugs the patient is taking and how well all of the pharmacological agents will be cleared so as to eliminate possible toxic interactions between the chemotherapy and the onboard drugs [47-49]. In summary, the clear increase in the global number of elderly, coupled with the concomitant later-life changes giving rise to increasing cancer rates and the potential age-related changes in the treatment of these cancers makes it essential that we develop models that can assist in our understanding of how normal aging cells and cancer cells interact.

## Brief Overview of the Core Model - Model 1

A detailed discussion of the ideas behind the model can be found in [14]. We briefly summarize that discussion in the following section.

### Introduction

A variety of papers, in the experimental literature, can be found to document the difference in the growth and/or proliferation rates of normal versus malignant cell lines. In particular, it is known that malignant cells can affect the growth/proliferation of surrounding normal cells. Further, the literature exhibits experimental data pointing to the fact that conjoint cultures of normal and neoplastic cells can be demonstrated to offer evidence for both the inhibition and stimulation of normal cells by these same conjoint neoplastic cells. Evidence for stimulation may be found in [50-54]. Evidence for inhibitory effects may be found in [55] and intermediate results are demonstrated by [56]. Rounds(1970) [57] demonstrates the existence of a growth modification factor which stimulates fibroblastic growth at low concentrations, but stops mitosis and is cytotoxic at high concentrations. We summarize these results as follows. There is a growth modification factor(GMF) released by a number of malignant human cell lines. This GMF has the following properties:

• At very low concentrations it does not affect fibroblast-like cells,

• At intermediate concentrations it can stimulate mitotic activity,

• At higher concentrations it can inhibit mitotic activity and finally,

• At very high concentrations it can kill surrounding fibroblast-like cells.

A possible relationship between cancer development, metastasis, and the surrounding normal cells may be hypothesized in the following manner. Suppose that there exists a tumor cell which is releasing GMF into the surrounding population of conjointly growing normal cells. Suppose further that, due to some factor (epigenetic, environmental, immune deficiency, or aging factors), the tumor cell divides (is not inhibited by the normal inhibitory processes of the surrounding *normal *cells), and therefore, it produces another cell which will subsequently increase the GMF density.

It is well known that normal cells can, if the conditions are correct, control the dynamics of tumor cells. That is to say, it is possible for a collection of normal cells which surround a single tumor cell, or small number of tumor cells, to control that cell or cells and to keep their growth restrained. It is hypothesized that this might occur through interference with the mitotic phase of the tumor cells. Such effects might occur through secretion of Pardee-like labile proteins [6,9,58,59]. Suppose, however, that the surrounding normal cells are unable to control the tumor cell population. Such an instance might occur, in aging tissue, when immune function has decreased and the tumor masking proteins are subsequently more effective. The inability of the normal cells to detect the tumor cells will cause a subsequent increase in the GMF titer around the developing tumor cell mass. As the titer of the GMF increases, the surrounding normal cells are killed due to the cytotoxic nature of high GMF concentrations. This toxic action makes room for subsequent divisions of the tumor cell population. In a region surrounding the tumor cell mass, but far enough away that the GMF titer is not at the toxic level, the fibroblasts are stimulated to form a surrounding boundary layer.

Several research groups have studied the growth and control of tumors from different perspectives via mathematical and theoretical modeling [60-67]. In the study of various therapeutic strategies such as chemotherapy, the major goal is to maximize the success of treatment. Therefore, in order to approach this goal, it is of critical importance to know the behavior and operation of the system that is under the influence of a given drug.

It has been proved that in a system comprised of normal and tumor cells, the development and growth of one component is not independent of the other. In particular, clinical evidence shows that the growth of tumor and normal cells is actually correlated each to the other [14,57,68,69]. The concept of the *growth modification factor *(GMF) and the conjoint growth of normal and tumor cells, was first mathematically introduced by Witten [6,9,12,13]. In this model, both the normal and the tumor cells increase according to a logistic growth law. However, the growth of normal cells *N *(*t*) is modified by an extra term *f*_*N*_(*T*), which is dependent upon the tumor cell population size. This model, derived from both clinical and experimental data, serves as a core model that can be used to explain the stimulation or inhibition of normal cells [5,14]. This is expressed as follows(1)(2)

where *T*, *N*, *K*_*T*_, *K*_*N*_, *r*_*T*_, *r*_*N *_are the total number of tumor cells, the total number of normal cells, the critical size of the tumor cells, the carrying capacity for the tumor cells, the carrying capacity for the normal cells, the per capita growth rate for the tumor and normal cells, and *f*_*T*_(*N*), *f*_*N*_(*T*) are the functional rules relating normal-to-tumor and tumor-to-normal interaction respectively [13]. Note that the previous logistic growth rule (equations (1a, 1b)) is a special case of the generalized logistic equation [70] given by(3)

where *ν *> 0 and *ν *→ 0 is understood as a limit and taking the limit gives the traditional Gompertz equation while *ν *= 1 yields the logistic equation. Our more generalized core model is then expressed by the following equation set:(4)(5)

Equations (1d)-(1e) provide a generalized growth-interaction model that may serve to explain the effects of GMF on the behavior of this conjoint aging-tumor cell population mixture. The role of the GMF factor is also crucial when the coupled system of normal and tumor cells goes under a chemotherapeutic treatment. The principle aim of this study is to quantitatively expand Witten's model during the course of chemotherapy. How then do we choose the two rules *f*_*N*_(*T*) and *f*_*T*_(*N*)?

The original core model (*ν *= 1) [13], expresses one possible dynamics for the interplay of normal and tumor cells as follows:(6)(7)

where *β *has the units of 1/time and *ρ*_0 _has units of cells. We will investigate this model as a first step in our discussion.

The tumor cells can only be affected by the normal cells up to a certain point. After that, there is a constant effect. To represent this behavior, Witten [13] chose a simple saturation function. One could replace this rule with a Hill function of degree *m *and easily discuss the behavior of that system as well. The tumor cell interaction with the normal cells is chosen as a logistic growth function. Again, alternative forms of interactive model may be chosen. For example, equation (2b) could be replaced with the generalized logistic growth model to yield the following equation (2b')(8)

However, for our initial discussion, we will consider the simple logistic growth equation in which  which reduces to our equation (2b). In the next section we discuss how this model may be modified to address chemotherapeutic intervention.

## Chemotherapeutic Modification and Simulation of the Core Model

Witten's model can be extended to address the medical scenario in which a conjoint cellular system interacts with a chemotherapeutic drug: *i.e.*, an elderly person undergoing chemotherapy. We assume that the drug kills both tumor cells and normal cells. The cellular response function to the pharmaceutical intervention can be mathematically structured as follows: *F *(*u*) = *a*_*i*_(1 - *e*^-*mu*^) where m is linked to the drug pharmacokinetics and is considered to be 1 in this preliminary study and *i *= *N, T*. In this expression, 1 - *e*^-*u *^represents the chemotherapy fractional cell kill and *u *is the amount of the drug at the tumor site at a specific time. The coefficient of *a*_*T *_and *a*_*N *_is the response coefficient factor of the tumor cells [71-73].

In this case, the core model 1 can be expressed by the following system of equations:(9)(10)

The last term shows the reduction in size of each cellular population as a function of the drug interaction in that population component. In subsequent sections we discuss the simulation of the evolution of both the normal and tumor cells for various interactions.

### 0.1 Untreated System Evolution

We first simulated the case when the system does not interact with the drugs (the drug terms in both equations are set zero). Figure [1] illustrates an example of how, for the chosen set of parameters, the conjoint effect of tumor cells and normal cells on each other can be seen. As the size of the tumor cells exceeds the critical size T*, which is here considered to be T* = 3.10^5^, the size of the normal cells *N *starts decreasing and the normal cells enter what we will call a inhibition phase in their population dynamics. The mini-window in the figure magnifies the behavior of the normal and tumor cells when the size of the tumor cells approaches the critical size of the tumor. In this figure the horizontal dashed line represents the critical size of the tumor cells. The system is arbitrarily considered to interact with the drug beginning at time *t *= 40.

### Evolution of a Treated System by Static Drugs

We now address the evolution of the normal and tumor cells when the drug is static (concentration of the drug is constant) and doesn't show a concentration diffusion over time. For this purpose, *u *and therefore *a*_*i*_(1 - *exp*(-*mu*)) are considered to be constants.

**Figure 2:**
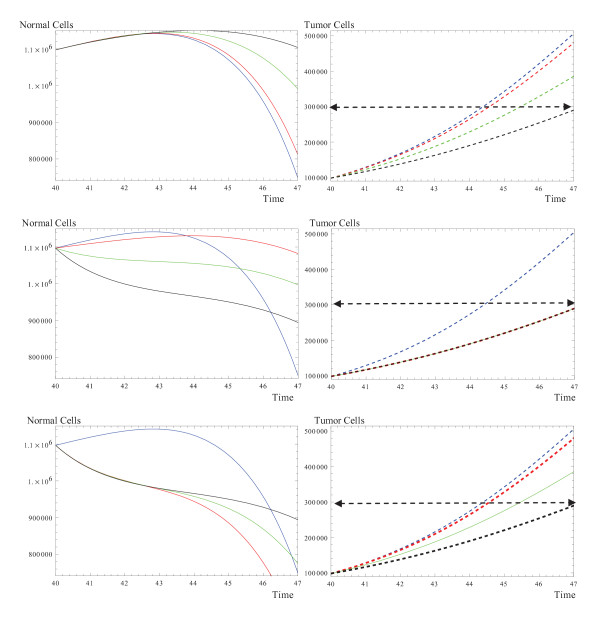
**The evolution of normal cells and tumor cells during the phase of therapy**. The drug is considered to be static. **First row: **the drug does not have any effects on normal cells, *a*_*N*_(1 - *e*^*mu*^) = 0, and *a*_*T*_(1 - *e*^*mu*^) = 0.01 (red), 0.05 (green), 0.1 (black). **Second row: **the drug kills both normal and tumor cells with more killing strength on the tumor cells. Blue represents the untreated system when *a*_*N*_(1 - *e*^*mu*^) = 0 = *a*_*T*_(1 - *e*^*mu*^) = 0. From there, the response of the tumor cells is considered to be constant, *a*_*T*_(1 - *e*^*mu*^) = 0.1, while a variation is considered for the response of normal cells as: *a*_*N*_(1 - *e*^*mu*^) = 0.01 (red), 0.05, (green), 0.1 (black). **Third row: **the drug kills both normal and tumor cells with more killing strength on normal cells. blue is untreated system when *a*_*N*_(1 - *e*^*mu*^) = 0 = *a*_*T*_(1 - *e*^*mu*^) = 0, From there, the response of the normal cells is considered to be constant, *a*_*N*_(1 - *e*^*mu*^) = 0.1, while a variation is considered for the response of the normal cells as: *a*_*T*_(1 - *e*^*mu*^) = 0.01 (red), 0.05, (green), 0.1 (black). The rest of the parameters are similar to the common parameter introduced in Figure [1].

In the first row of the Figure [2], the evolution of normal cells and tumor cells are simulated when the system interacts with a drug. It is assumed that the drug kills only tumor cells and has no effect on normal cells. As the effect of the drug increases, tumor cells show a slower growth in evolution. Therefore, their population size exceeds the critical tumor size later in time. Not only is this slower growth significant by itself, but the existence of the larger population of normal cells during the course of therapy is also distinguished. Furthermore, it is important that normal cells enter the phase of inhibition later as compared to the untreated normal cells in the untreated system.

The second row in Figure [2] examines the case where the drug kills both normal and tumor cells with more weight on killing the tumor cells. We have considered that the drug kills tumor cells with a specific strength. Considering this assumption, we study the system where the normal cells are killed with different strength. As the drug kills more normal cells, the seize of these cells decreases during the course of therapy, however, the normal cells enter the phase of inhibition with a delay because of the slower growth of the tumor cells caused by the drug's killing effect on tumor cells.

The last row in Figure [2] simulates the case where the drug kills both normal and tumor cells with more weight on killing the normal cells. The effect of the drug on normal cells is thought to remain the same, while a variation is considered for the death of tumor cells by the drug. As can be seen at the beginning of the therapy, normal cells experience the same decrease in their size, while they were split toward the end and, thus, enter the phase of inhibition at different times due to the different killing strength of the drug on tumor cells.

To summarize this section, we can see that in the untreated case, tumor cells growth fast and normal cells experience a sharp decay in their size. In the treated case, the size of the normal cells is initially maintained and the dropping behavior is delayed when just tumor cells are killed by the drug. When the drug kills both normal and tumor cells, but more tumor cells than normal cells, the decrease in the initial size of the normal cells can be detected together with a delay in entering the decaying phase.

### Evolution of a Treated System by Dynamic Drugs

**Figure 3:**
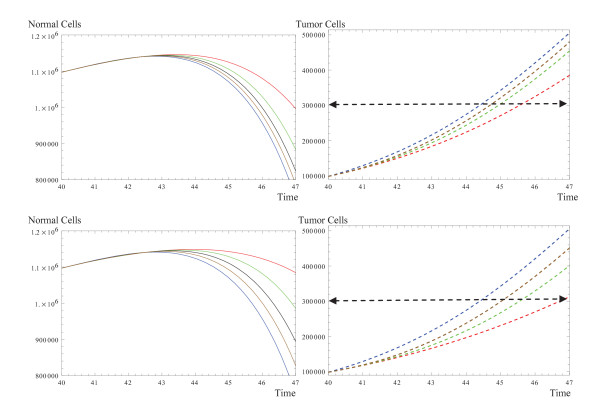
**The evolution of normal cells and tumor cells during the phases of therapy**. The drug is considered to be dynamic and its concentration diffuses exponentially over time. *u*_0 _is the initial value of the drug and *d *is the decaying rate, and *m *is linked to pharmacokinetics and considered to be 1 in this study. The drug does not have any effect on normal cells, *a*_*N*_(1 - *e*^*mu*^) = 0, *a*_*T *_= 0.1. **First row: **The evolution of normal and tumor cells is simulated for different drug decaying rates. *u*_0 _= 1, and untreated (blue), *d *= 0.1 (red), 0.5 (green), 1 (black), and 2 (brown). As can be seen, the system tends to behave as untreated as the decaying rate increases. **Second row: **Same parameters in the first row except the initial value of the drug is increased, *u*_0 _= 3, which maintains the diffusion behavior of the drug leading to slower growth for the tumor cells and a delay in entering the inhibition phase for the normal cells. The rest of the parameters are similar to the common parameter introduced in Figure [1].

In this section, the drug is considered to lose its strength exponentially over time. This behavior is expressed as: *u *= *u*_0_*exp*(-*d*·*t*) where *u*_0 _is the initial value of the drug and *d *is the decay rate. In the first row of figure 3, *u*_0 _is chosen to be 1, while the decaying rate is increased. As can be seen even in the presence of the drug, tumor and normal cells can show a behavior similar to that of an untreated system when the diffusion is large. In the second row, the increase in the decaying rate is combined with an increase in the initial value of the drug. In this case, the diffusion behavior is maintained and a more successful outcome in slowing down the growth of the tumor cells and a later transition to the inhibition phase for the normal cells is achieved. It should be mentioned that the decay in strength of the drug can be formated in a linear or a bell shaped decay as well. This will be studied elsewhere.

## Model 2 - Gompertzian Tumor Growth

A number of tumors have been demonstrated to follow what is called Gompertzian growth [74,75]. In this case, we would modify the tumor growth equation (2a) as follows, allowing the normal cell equation to remain as before:(11)(12)

where *h*_0 _and *γ *are parameters of tumor growth. Demicheli *et al. *[76] provide growth relationships and derive the two parameters for the LoVo tumor cell line.

**Figure 4:**
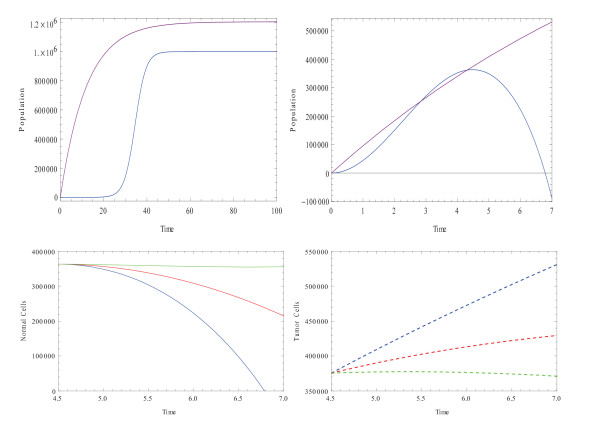
**Model 2**. **Top-Blue: **Evolution of normal cells, **Purple: **Evolution of tumor cells. Common parameters: *r*_*N *_= 0.4, *h *= 10^5^, γ = 0.083, *K*_*N *_= 10^6^. **Top left: **there is no interaction between normal cells and tumor cells *k *= 0, *β *= 0. Tumor cells show a higher increasing rate at the beginning compared to the normal cells. **Top right: **normal and tumor cells are allowed to interact, *k *= 1, *β *= 2, *ρ*_0 _= 1, *ρ*_1 _= 1000, T* = 3.10^5^, *N*_0 _= 1, *T*_0 _= 1. Fast growing tumor cells force the normal passes the critical size of tumor cells quickly and force normal cells enter to the inhibition phase in a short time. **Down: **The evolution of normal cells and tumor cells during the phase of therapy. The drug is considered to be static. **Down Left: **the drug does not have any effects on normal cells, *a*_*N*_(1 - *e*^*mu*^) = 0, and *a*_*T*_(1 - *e*^*mu*^) = 0.0 (Blue-untreated system), 0.1 (red), and 0.17 (green). Effect of the drug in slower growth of tumor cells and a delay in entering the inhibition phase for the normal cells can be detected.

Using the above equations, the behavior of tumor and normal cells is simulated in Figure [4]. In the first step, the evolution behavior is simulated when there is no inter-communication between the tumor and normal cells and also in the absence of any drugs. As can be seen in this figure, after a long period of time, the population size of the tumor cells is almost the same for the logistic growth as for the Gompertizan growth. However, the tumor cells initially show a higher increasing rate in the Gompertizan growth than in the logistic growth.

Considering the inter-connection coupling also seen, in Figure [4] (top right) the normal cells enter the inhibition phase very fast and start decreasing in a very short amount of time. This behavior is associated with the fact that tumor cells grow at a very fast rate and, thus, exceed critical size in a short amount of time.

The evolution of a system treated by static drugs is also simulated in Figure [4]. In the simulation, it is considered that the drug ideally kills just the tumor cells and has no effect on the normal cells, *a*_*N *_= 0. The strength of the drug is considered to begin at *a*_*T *_(1 - *e*^*mu*^) = 0.1 and increase to *a*_*T*_(1 - *e*^*mu*^) = 1. Returning to Figure [2] (top left), the size of the normal cells at the beginning of chemotherapy is almost 1.1*10^6^. In the absence of any treatments, this size drops below 8*10^5^. However, by interacting with an anti-tumor drug with the strength of 0.1, the normal cells experiences no drop in size.

In Model 2, the therapy begins once the normal cells enter the inhibition phase, exactly like Model 1. In this case as can be seen in Figure 4 (below, left), the size of the normal cells is almost 3.6*10^5 ^at the beginning of the therapy. With no treatments, they soon decay to zero. Implementing the drug with a strength of *a*_*T*_(1 - *e*^*mu*^) = 0.1 maintains the size of the normal cells at 2.2*10^5 ^by the end of the therapy.

A drug with the strength of *a*_*T*_(1 - *e*^*mu*^) = 0.17 suppresses the dropping behavior of normal cells. Based on the results of the simulation, it is evident that in order to block the decreasing behavior of normal cells a stronger drug with more power to kill tumor cells is needed in the Gompertizan tumor growth, seen in Model 2, than in the logistic tumor growth, as shown in Model 1. The behavior of the tumor cells during the therapy is also simulated above. The decrease in the size of tumor cells for two different strengths of 0.1 and 0.17 can be seen in figure 4 as well.

## Closing Remarks

This work was based on a modification of Witten's conjoint tumor cell-aging fibroblast cell model [13]. The conjoint evolution of both a normal and a tumor cell population were studied both with and without the effects of an interacting hypothetical chemotherapeutic drug.

In the absence of any drugs, the growth dynamics of the individual populations of normal and tumor cells is not independent due to the biochemical cross-talk and biomechanical interactions. In fact, the relative growth and size of the tumor/normal cell populations can control the opposing population. We note that as the size of the tumor cells exceeds a hypothetical critical size T*, the normal cells can no longer control the tumor cell population size and the population of normal cells eventually goes extinct.

The evolution of conjoint normal and tumor cell populations were then studied under the influence of chemotherapeutic drugs, and also by pre-setting model parameters such as *β*, *ρ*, and *k*. The system can be shown to illustrate a variety of different behaviors under a different choices of the model parameters. As the drug kills more tumor cells than normal cells, the tumor cells approach the critical size more slowly. This generates a delay in the decline of the normal cell population. Such a mechanism might allow for mixed therapeutic intervention such as joint radiation and chemotherapy. Further analysis needs to address how the model parameters might change over the chronological age of the patient and how this would affect the results of the chemotherapeutic intervention.

We conclude that the behavior of the system is complex and that the specifications of a chosen drug in terms of the decay and initial value, combined with the specifications of the system in terms of the inter-dependence of compartments during their evolution, are all critical factors shaping the behavior of the system during therapy. This knowledge may introduce a path to advance the treatment of age-related tumor development and treatment.

Future work may include the effect of the drug determining the effect of time delay in absorption at the tumor cite, or considering a kind of multiple therapy, which would combat cancer by focusing on the importance of immunotherapy and the strengthening the immune system.

## Methods

### Computational Calculations

All calculations were executed on an PC using Mathematica v6.0. Code is available from the first author.

## Competing interests

The authors declare that they have no competing interests.

## Authors' contributions

The original conjoint cell culture equations were drawn from earlier work of TMW. Modifications for therapeutic intervention were made by ASF. Computational work was carried out principally by the first author with suggestions from the second. All other work was executed jointly. There was no funding for this research project. All authors have read and approved the final manuscript.
